# 

**Published:** 1876-01

**Authors:** 


CONTENTS:

| | PAGE | | PAGE |
|---|---|---|---|
| HALL'S JOURNAL FOR 1876 | 1 | GETTING MARRIED | 18 |
| POPULAR PREJUDICES | 3 | TIGHT SHOES | 26 |
| LIFTING MACHINE | 5 | GLUTTONY | 28 |
| A WICKED THEFT | 5 | DISEASE AND CRIME | 28 |
| GENUINE ENTERPRISE | 7 | BODILY CARRIAGE | 31 |
| HEAT AND COLD | 8 | TRAINING SCHOOL | 34 |
| WHOLESOME FOODS | 9 | LIFE ON RAIL ROAD | 37 |
| INTERESTING TO ALL | 10 | SUICIDE | 39 |
| SLEEPING IN CHURCH | 11 | COMMON SENSE | 42 |
| WINTER DISEASES | 11 | INSTINCT OF APPETITE | 44 |
| DEATH IN-DOORS | 13 | SLEEP DELICIOUS | 46 |
| AIRING CHAMBERS | 14 | A GOOD DIGESTION | 47 |
| NOTHING TO DO | 17 | SEA VOYAGES | 48 |

